# Do the Competitions Played During Congested Weeks Influence the External Load of Spanish Soccer Teams? Analysis by Match Playing Time

**DOI:** 10.1186/s40798-026-01008-x

**Published:** 2026-03-18

**Authors:** David Lobo-Triviño, Tomás García-Calvo, Jorge Polo-Tejada, Javier Raya-González, Roberto López del Campo, Ricardo Resta, Aldo A. Vasquez-Bonilla

**Affiliations:** 1 Faculty of Sport Sciences, University of Extremadura, C/ Avenida de la Universidad, s/n, C.P., 10003 Cáceres, Spain; 2 Research Group on Sport and Physical Education for Personal and Social Development (GIDEPSO), Department of Specific Didactics, Faculty of Education Sciences and Psychology, University of Córdoba, Córdoba, Spain; 3 Department of Competitions and Mediacoach, LaLiga, Madrid, Spain

**Keywords:** Football, Fixture, Match running performance, Player participation, Elite

## Abstract

**Background:**

This study aimed to examine differences in external load among professional soccer players during congested weeks across different competition types (League, Cup, and Champions League (UCL)), considering individual match playing time. A retrospective, descriptive, and longitudinal analysis was conducted using external load data from 120 players belonging to the top five teams in the Spanish LaLiga during the 2023/24 season, including 2,671 match observations. Players were categorised by playing time (Starters, Replaced, Fringe, and Non-Starters), and weeks were classified as non-congested (NCON) or congested (CON), with further distinction by competition type. External load metrics were recorded using the Mediacoach^®^ tracking system and analysed through Linear Mixed Models.

**Results:**

Results indicated that players in NCON weeks covered significantly less distance at very low-speed running (VLSR) and at low-speed running (LSR) compared to CON weeks (both *p* < .01). Players in CON weeks by League covered significantly higher total distance (TD) compared to UCL congested weeks (*p* < .001) and NCON weeks (*p* < .01). In CON weeks by Cup, Non-Starters, who accumulated between 0 and 59 min across two matches, covered significantly higher very high-speed running (VHSR) than CON weeks by UCL (*p* < .05) and NCON weeks (*p* < .05).

**Conclusions:**

These findings underscore the relevance of competition context and match playing time when analysing external load during matches. Tailoring training and rotation strategies based on external load data and competition type may help maintain performance and reduce fatigue.

**Supplementary Information:**

The online version contains supplementary material available at 10.1186/s40798-026-01008-x.

## Key Points

- Participation or non-participation in a midweek competition results in variations in the external load recorded during the subsequent weekend match.
- The external load observed in the weekend match is affected by the type of competition played in the middle of the week.
- Managing players’ playing time helps optimise their performance in the following match.
- Implementing optimal recovery strategies for players is essential when matches are scheduled within short time intervals.

**Supplementary Information:**

The online version contains supplementary material available at 10.1186/s40798-026-01008-x.

## Background

Over the past decade, elite soccer has placed increasing physical demands on players during matches, largely due to the intensification of European competitions and the globalised nature of modern soccer [1]. Furthermore, fluctuations in external load are common, particularly in response to congested (CON) fixture periods [2], which have been increasing over the last decade [3]. These periods are defined as the accumulation of matches within a short period, typically with less than 96 h between official matches [4], faced by top-tier European clubs [3, 5], including the top five LaLiga clubs (Spanish First Division).

Top soccer clubs may face up to six matches over an 18-day span during certain phases of the season, imposing a substantial physiological and psychological burden on players [6]. In this context, it is important to note that declines in match external load may be especially pronounced during congested fixture periods [7]. This decrease is likely associated with increased neuromuscular fatigue and compromised recovery processes [8]. As a consequence, players may face a higher risk of injury, which can negatively affect both team performance and individual well-being [9]. Although existing evidence highlights these issues, there is no research analysing how the different competitions faced by top-tier Spanish LaLiga clubs during congested weeks, such as the Champions League (UCL) and the National Cup (Cup), interact and how this interaction influences external load demands. These tournaments might impose different external load demands but may also induce higher levels of motivation and stress due to factors such as the prestige of the match, the team’s tactical priorities, required travel, and available recovery time [10]. Thus, a competition-specific analysis becomes essential for a deeper and more accurate understanding of the effects in CON fixture.

Moreover, during tournaments, it is common for key players to accumulate substantial playing time across consecutive matches, often participating in national league fixtures as well as in Cup or UCL knockout matches within the microcycle (i.e., within a single training week). For instance, some starting players in Spanish LaLiga teams can exceed 200 min of play in a single week, particularly during the decisive phases of the season [11]. Some external load indicators, such as high-speed running (HSR), sprints, and total distance (TD) covered, could be affected by sustained player participation. For example, dos Santos Guimarães et al. [2] showed that players who had played more than 110 min in congested weeks covered more HSR than those who played less than 50 min. However, few studies have examined how these outcomes vary as a function of both match playing time and the specific competition involved in each CON fixture. Given the match running performance variability due to competition context, there is a need for a study that integrates both match playing time and the type of the competition within each CON fixture.

Unlike previous studies, which typically treat congested matches as homogeneous blocks without considering the competition type or the individual time exposure of each player, this study includes three key advances. Firstly, the analysis was conducted across an entire season, which may reduce match-to-match variability. Secondly, it differentiates between non-congested (NCON) and CON fixtures based on the type of competitions involved (such as League, Cup and UCL), recognising that each competition presents distinct contextual demands. Thirdly, the individual participation of each player is included, acknowledging that accumulated fatigue is not the same for all players. For example, players who accumulate 170 min over two matches may experience a different physical impact than those who play only 60 min [12]. The inclusion of these three factors provides a more nuanced understanding of how CON periods affect physical performance in elite soccer and better capture the interaction between competitions. Therefore, the main objective of this study was to examine the influence of competition type (i.e., Cup, League and UCL) and match playing time on external load during CON weeks in top five Spanish soccer teams. Our hypothesis is that distances covered at low-intensity will be greater in CON weeks compared to NCON weeks [7]. Related to this, in terms of match playing time, substitute players will have higher relative values in the variables of distance covered at high-intensity and sprint distance [13].

## Methods

### Study Design

A retrospective, descriptive, and longitudinal study was conducted to examine the differences in external match load between CON and NCON weeks according to the type of competition, considering the match playing time of soccer players. The external load during the experimental period was recorded through the Mediacoach^®^ system. Weeks were classified into non-congested (NCON; i.e., one match per week) and CON (i.e., two matches per week). Furthermore, a distinction was made between weeks that were congested by National Cup (Cup), LaLiga (League) or Champions League (UCL) matches, which were played midweek (usually Tuesday, Wednesday, or Thursday). Therefore, all values of external load belonged only to the official matches from LaLiga played over weekend. Additionally, players were classified according to their playing time in the match or matches from the same week based on Anderson et al. [14]: Starters, players who played more than 150 min in total; Replaced, players who played between 95 and 149 min in total; Fringe, players who played between 60 and 94 min in total; Non-Starters, players who played between 0 and 59 min in total. However, during NCON weeks, soccer players were only classified as Fringe and Non-Starters players since it is not possible to exceed the 94-minute threshold in a single match.

### Participants

120 professional soccer players from the top five teams in the Spanish LaLiga who competed in the League, Cup and UCL competitions were included in the study during the 2023/24 season. Therefore, the sample consisted of 2,671 individual observations from 108 matches. Goalkeepers were not included in the analysis due to their specific role during the game. Data were provided by LaLiga™. The study was approved by the Bioethics and Biosafety Commission of the University of Extremadura’s Vice-Rectorate for Research, Transfer, and Innovation (protocol number: 79//2025), and was conducted in accordance with the Declaration of Helsinki (2013).

### Procedure

External load data were collected using a multi-camera tracking system known as Mediacoach^®^. This system assesses the distance covered by teams (in metres) and the number of high-intensity actions and sprints (LaLiga™, Madrid, Spain). It features a series of super 4 K High Dynamic Range cameras equipped with a positioning system (Tracab-ChyronHego VTS) that records and analyses the X and Y positions of each player from multiple angles, providing real-time three-dimensional tracking. The tracking data are recorded at a rate of 25 Hz. The validity and reliability of this system for the selected variables have been previously assessed [15–17], demonstrating strong correlations (*r* > .80) and high intraclass correlation coefficients (ICC > 0.75) between the Mediacoach multi-camera tracking system and the Global Positioning System.

### Study Variables

External load was divided into the following categories: total distance covered by players (TD), distance covered between 0 and 6 km·h^− 1^ (very low-speed running, VLSR), distance covered between 6 and 12 km·h^− 1^ (low-speed running, LSR), distance covered between 12 and 18 km·h^− 1^ (medium-speed running, MSR), distance covered between 18 and 21 km·h^− 1^ (high-speed running, HSR), distance covered between 21 and 24 km·h^− 1^ (very high-speed running, VHSR), distance covered between 24 and 28 km·h^− 1^ (Sprint), distance covered above 28 km·h^− 1^ (High Sprint), number of accelerations above 3 m·s^− 2^ (ACC), number of decelerations above − 3 m·s^− 2^ (DEC), average intensity of the accelerations performed in the match, measured in m·s^− 2^ (ACC_AVG_), average intensity of the decelerations performed in the match, measured in m·s^− 2^ (DEC_AVG_), maximum speed achieved in the match (Max. Speed), number of sprints (> 24 km·h^− 1^, Nº Sprint), distance covered with a power consumption above 25.5 W·kg^− 1^ (HMLD). The values of this last variable correspond to running at a constant velocity of 5.5 m·s^− 1^ or 19.8 km·h^− 1^ on grass, accelerations or decelerations (e.g., accelerating from 2 to 4 m·s^− 2^ for 1 s) also are included in HMLD [18]. All variables, except for ACC_AVG_, DEC_AVG_, and Max. Speed, were normalised to metres or number of actions per unit of time (m/nº · min^− 1^).

### Statistical Analysis

All statistical analyses were performed using RStudio version 2025.09.0 + 387 [19]. Given the hierarchical structure of the data, with repeated observations nested within individual players and the longitudinal nature of the dataset, Linear Mixed Models (LMMs) were employed as the primary analytical approach. LMMs are particularly suited for handling unbalanced designs and repeated measures, making them an appropriate choice for this study, where external load variables are nested within individual players [20]. Each player’s dataset included multiple match records, with observations from several players per match.

To properly account for the cross-classified and multi-level structure of the data, a multi-level modelling strategy was implemented, incorporating both fixed and random effects. A two-level hierarchical framework was established: Level 1 represented repeated measures across matches, while Level 2 accounted for individual player variability. The dependent variables in our models included external workload metrics, such as distances covered at different speed thresholds. The independent variables consisted of week types (NCON, Cup, League, and UCL) and match playing time categories (Starters, Replaced, Fringe, and Non-Starters), modelled as fixed effects. To capture individual variability, player identity was included as a random effect. Model estimates were reported as coefficients with standard errors (Coeff ± SE), with statistical significance set at *p* < .05.

Intraclass correlation coefficients (ICC) values were calculated from unconditional models using the ratio between player-level variance and total variance. ICCs ranged approximately from 0.33 to 0.58 for distance-based variables, indicating a meaningful proportion of variance attributable to the player level.

## Results

### The Influence of Congested Schedule on External Load

Tables 1 and 2 show the differences in external load between CON and NCON weeks. Players in NCON weeks covered significantly less distance at VLSR and LSR than CON weeks (*p* < .01).

**Table 1:** Differences in distance related variables between CON and NCON weeks

	TD (m·min^− 1^)	*p*	VLSR (m·min^− 1^)	*p*	LSR (m·min^− 1^)	*p*	MSR (m·min^− 1^)	*p*	HSR (m·min^− 1^)	*p*	VHSR (m·min^− 1^)	*p*	Sprint (m·min^− 1^)	*p*	High Sprint (m·min^− 1^)	*p*
	Coeff (SE)		Coeff (SE)		Coeff (SE)		Coeff (SE)		Coeff (SE)		Coeff (SE)		Coeff (SE)		Coeff (SE)	
CON (Intercept)	121.67		35.43	**	40.57	**	32.93		8.11		4.68		2.81		0.87	
NCON	− 0.33 (0.35)		− 0.51 (0.17)		− 0.55 (0.21)		0.01 (0.21)		0.06 (0.08)		0.05 (0.06)		0.08 (0.05)		0.02 (0.03)	

Coeff  = Coefficient, SE = Standard Error, CON = Congested week, NCON = Non-congested week, TD = Total distance; VLSR = distance covered between 0–6 km·h^− 1^; LSR = distance covered between 6–12 km·h^− 1^; MSR = distance covered between 12–18 km·h^− 1^; HSR = distance covered between 18–21 km·h^− 1^; VHSR = distance covered between 21–24 km·h^− 1^; Sprint = distance covered between 24–28 km·h^− 1^; Max. Sprint = distance covered above 28 km·h^− 1^; **p < .01

**Table 2:** Differences in accelerations, decelerations and sprint variables between CON and NCON weeks

	ACC	*p*	DEC	*p*	ACC_AVG_	*p*	DEC_AVG_	*p*	Max. Speed	*p*	Nº Sprint	*p*	HMLD	*p*
	Coeff (SE)		Coeff (SE)		Coeff (SE)		Coeff (SE)		Coeff (SE)		Coeff (SE)		Coeff (SE)	
CON (Intercept)	22.62		22.95		0.75		− 0.74		30.41		0.24		32.35	
NCON	− 0.09 (0.09)		− 0.08 (0.09)		− 0.00 (0.00)		0.00 (0.00)		− 0.03 (0.07)		0.00 (0.00)		0.11 (0.20)	

Coeff = Coefficient, SE = Standard Error, CON = Congested week, NCON = Non-congested week; ACC = number of total high accelerations per minute; DEC = number of total high decelerations per minute; ACC_AVG_ = average intensity of the accelerations performed in the match, measured in m/s^2^; DEC_AVG_ = average intensity of the decelerations performed in the match, measured in m/s^2^; Max. Speed = maximum speed achieved in the match; Nº Sprint = number of sprints performed per minute (> 24 km·h^− 1^); HMLD = distance covered with a power consumption above 25.5 W·kg-1 per minute

### Differences on External Load According to Competition Type

**Fig. 1:**
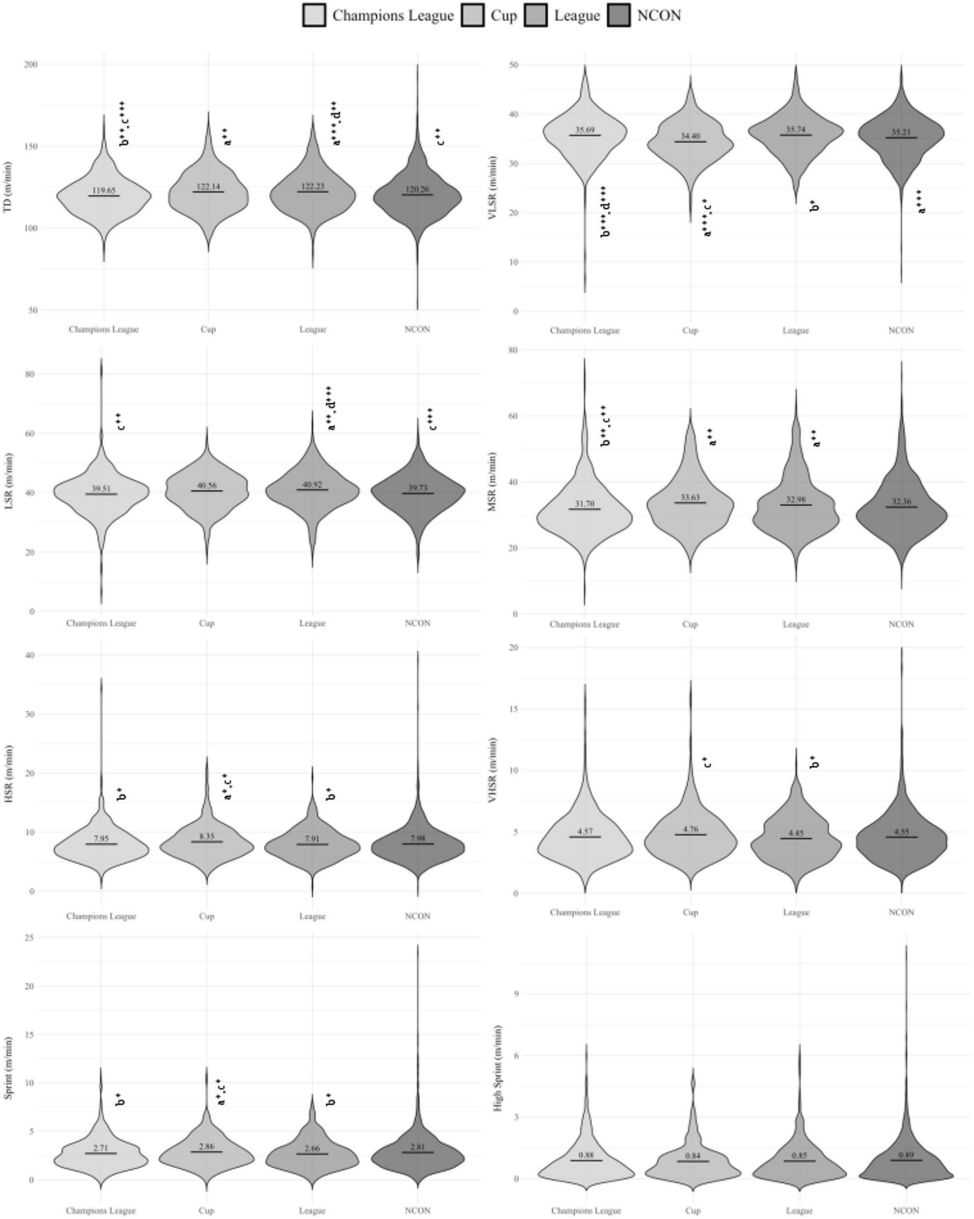
Differences on TD and distance at different speeds thresholds between different competition type (Champions League, Cup, League and NCON). a = significant differences with respect to Champions League week; b = significant differences with respect to Cup week; c = significant differences with respect to League week; d = significant differences with respect to Non-congested week; *p < .05; **p < .01; ***p < .001

**Fig. 2:**
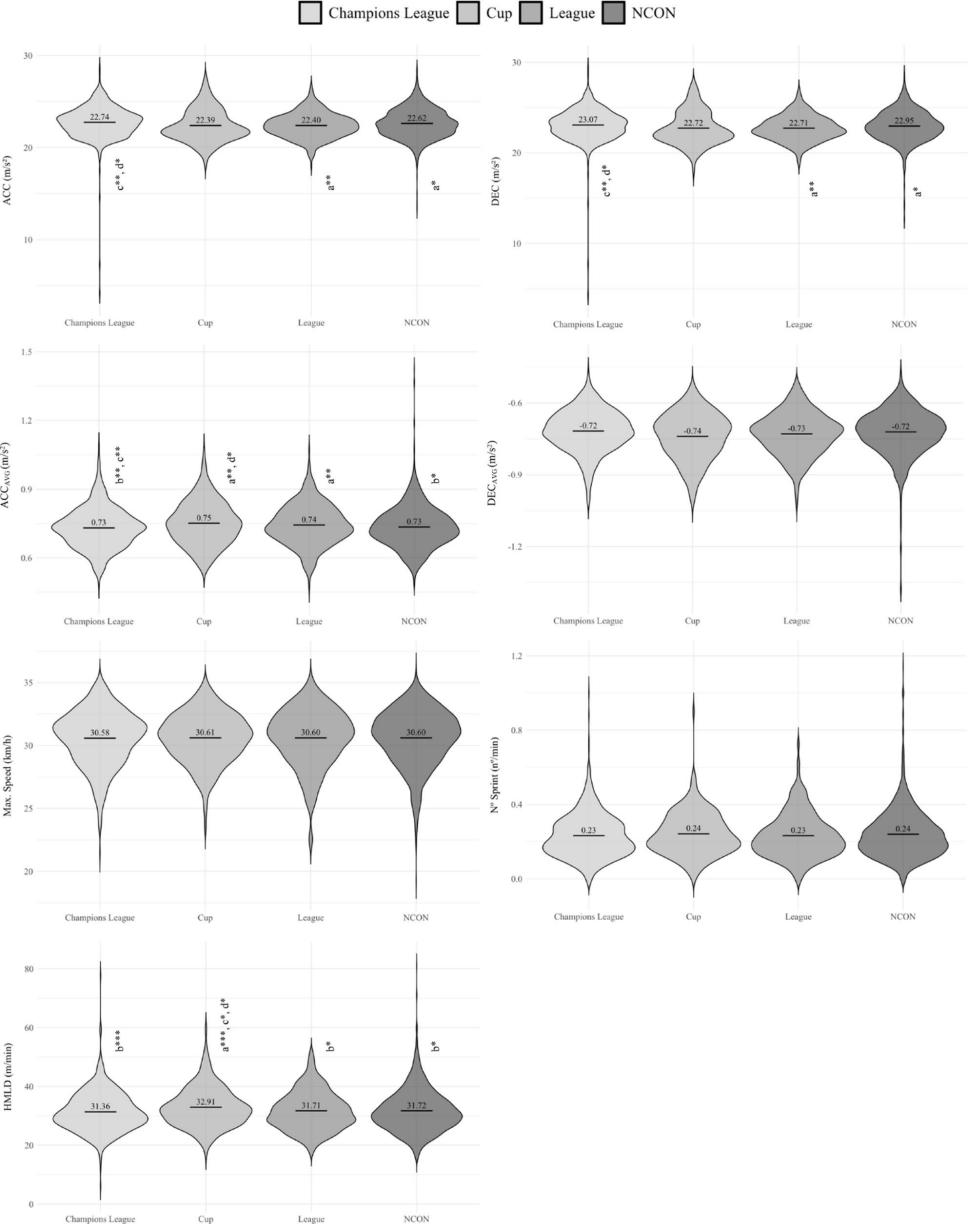
Differences in acceleration, deceleration and sprint variables between different competition type (Champions League, Cup, League and NCON). a = significant differences with respect to Champions League week; b = significant differences with respect to Cup week; c = significant differences with respect to League week; d = significant differences with respect to Non-congested week; *p < .05; **p < .01; ***p < .001

Figures 1 and 2 depict the differences in external load according to competition type (UCL, Cup, League and NCON). Players in CON weeks by UCL covered significantly less TD than in CON week for Cup (*p* < .01) and League (*p* < .001). However, in the weeks CON by UCL, players achieved higher ACC values compared to congested weeks by League (*p* < .01) and NCON (*p* < .05) weeks.

These results highlight that CON weeks by UCL are associated with lower total distance but increased acceleration, suggesting a different intensity profile compared to other competition types.

### Differences on External Load According to Competition Type and Match Playing Time

The analysis of TD and distance at various speed thresholds across competition types (UCL, Cup, League and NCON) according to match playing time (i.e., Starters, Replaced, Fringe and Non-Starters players), is presented in Table 3.

**Table 3:** Differences in distance related variables between players with different match participation according to congestion type

		Starters	*p*	Replaced	*p*	Fringe	*p*	Non-Starters	*p*	*Between players comparison*
		Coeff (SE)		Coeff (SE)		Coeff (SE)		Coeff (SE)		
TD (m·min^− 1^)	UCL	118.67 (1.02)		120.73 (1.01)		121.54 (1.33)		120.83 (1.19)	+**, µ*	
	Cup	120.23 (1.33)		122.25 (1.20)		123.07 (1.87)		128.36 (1.79)	#**, ø*	c**, e*
	League	121.04 (1.08)		122.22 (1.05)		125.57 (1.52)		126.17 (1.46)	#*	c*
	NCON	-		-		121.78 (0.95)		122.94 (0.90)	+*	
VLSR (m·min^− 1^)	UCL	35.80 (0.40)		36.83 (0.40)	+**	35.53 (0.58)		34.61 (0.50)		e**
	Cup	34.94 (0.58)		34.69 (0.51)	#**	34.77 (0.86)		32.47 (0.82)		
	League	35.86 (0.44)		35.80 (0.42)		34.33 (0.68)		34.31 (0.65)		
	NCON	-		-		35.29 (0.36)		34.20 (0.33)		f*
LSR (m·min^− 1^)	UCL	39.36 (0.55)		40.82 (0.54)		41.10 (0.76)		39.37 (0.67)		
	Cup	40.04 (0.76)		40.78 (0.67)		40.92 (1.11)		41.63 (1.05)		
	League	40.80 (0.59)		41.04 (0.57)		42.09 (0.88)		41.94 (0.84)		
	NCON	-		-		40.99 (0.50)		39.95 (0.47)		
MSR (m·min^− 1^)	UCL	30.98 (0.67)		32.68 (0.66)		32.55 (0.84)		32.74 (0.77)	+***, µ*	a*
	Cup	31.98 (0.84)		33.23 (0.77)		33.78 (1.16)		37.86 (1.11)	#***, ø*	c***, e**
	League	32.17 (0.70)		32.84 (0.69)		35.38 (0.95)	ø*	35.68 (0.92)	#*	b*, c**, e*
	NCON	-		-		32.62 (0.63)	µ*	34.34 (0.60)	+*	f***
HSR (m·min^− 1^)	UCL	7.47 (0.22)		8.09 (0.22)		8.01 (0.30)		8.71 (0.26)		c***
	Cup	7.79 (0.29)		8.36 (0.26)		8.49 (0.43)		9.69 (0.41)		c**
	League	7.51 (0.23)		7.91 (0.23)		8.43 (0.34)		8.87 (0.33)		c**
	NCON	-		-		7.94 (0.20)		8.68 (0.19)		f***
VHSR (m·min^− 1^)	UCL	4.36 (0.16)		4.70 (0.16)		4.63 (0.22)		4.93 (0.19)	+*	
	Cup	4.73 (0.21)		4.71 (0.19)		4.61 (0.31)		6.05 (0.29)	#*, ø*	c**, e**, f**
	League	4.28 (0.17)		4.49 (0.17)		4.79 (0.25)		5.05 (0.24)		
	NCON	-		-		4.51 (0.15)		5.11 (0.14)	+*	f***
Sprint (m·min^− 1^)	UCL	2.57 (0.14)		2.78 (0.13)		2.67 (0.18)		2.97 (0.16)		
	Cup	2.98 (0.18)		2.90 (0.16)		3.04 (0.26)		3.38 (0.25)		
	League	2.59 (0.14)		2.67 (0.14)		2.89 (0.21)		3.10 (0.20)		
	NCON	-		-		2.69 (0.12)		3.22 (0.12)		f***
High Sprint (m·min^− 1^)	UCL	0.80 (0.07)		0.94 (0.07)		0.74 (0.10)		0.93 (0.09)		
	Cup	0.85 (0.10)		0.85 (0.09)		1.10 (0.15)		0.84 (0.14)		
	League	0.81 (0.08)		0.85 (0.08)		1.00 (0.12)		0.77 (0.11)		
	NCON	-		-		0.84 (0.06)		0.97 (0.06)		

Coeff  = Coefficient, SE = Standard Error; Starters = players who played more than 150 min; Replaced = players who played between 95–149 min; Fringe = players who played between 60–94 min; Non-Starters = players who played between 0–59 min; TD = Total distance; VLSR = distance covered between 0–6 km·h^− 1^; LSR = distance covered between 6–12 km·h^− 1^; MSR = distance covered between 12–18 km·h^− 1^; HSR = distance covered between 18–21 km·h^− 1^; VHSR = distance covered between 21–24 km·h^− 1^; Sprint = distance covered between 24–28 km·h^− 1^; High Sprint = distance covered above 28 km·h^− 1^; UCL = congestion caused by Champions League match; Cup = congestion caused by Cup match; League = congestion caused by League match; NCON = week in which only one match is played, non-congested week; # = significant differences respect to UCL; + = significant differences respect to Cup; µ = significant differences respect to League; ø = significant differences respect to NCON; a= significant differences between Starters and Replaced; b = significant differences between Starters and Fringe; c = significant differences between Starters and Non-Starters; d = significant differences between Replaced and Fringe; e = significant differences between Replaced and Non-Starters; f = significant differences between Fringe and Non-Starters; *p < .05; **p < .01; ***p < .001

Total distance (TD): Non-Starters players covered significantly higher TD in CON weeks with Cup compared to UCL congestion (*p* < .01) and NCON (*p* < .05) weeks.

Very low-speed running (VLSR): In CON weeks by UCL, Replaced players significantly increased values compared to Cup congestion (*p* < .01).

Medium-speed running (MSR): In CON weeks by League, Fringe players covered significantly higher distance compared to NCON weeks (*p* < .05). For Non-Starters players, MSR was significantly increased in CON weeks with Cup compared to UCL (*p* < .001) and NCON (*p* < .05) weeks.

Very high-speed running (VHSR): In CON weeks by Cup, Non-Starters players covered significantly more distance compared to UCL congestion (*p* < .05) and NCON (*p* < .05) weeks.

No group of players experienced significant differences in distance covered at Sprint and High Sprint between competition types.

Between players comparison: During NCON weeks, Non-Starters players covered significantly more distance MSR, HSR, VHSR and Sprint distance than Fringe players. Similarly, during the CON weeks by Cup, VHSR was significantly higher for Non-Starters compared to all other players (*p* < .01).

These findings underscore the importance of playing time status, as Non-Starters generally show greater high-intensity efforts compared to other player categories, particularly during weeks CON by Cup.

The differences in acceleration, deceleration and sprint variables between different competition types (UCL, Cup, League and NCON) according to match playing time (i.e., Starters, Replaced, Fringe and Non-Starters players) are presented in Table 4.

**Table 4:** Differences in acceleration, deceleration and sprint variables between players with different match participation according to competition type

		Starters	*p*	Replaced	*p*	Fringe	*p*	Non-Starters	*p*	*Between players comparison*
		Coeff (SE)		Coeff (SE)		Coeff (SE)		Coeff (SE)		
ACC	UCL	22.66 (0.18)		23.12 (0.18)		22.55 (0.29)		23.02 (0.25)		
	Cup	22.60 (0.29)		22.53 (0.25)		22.22 (0.46)		22.14 (0.44)		
	League	22.41 (0.20)		22.44 (0.19)		22.22 (0.35)		22.03 (0.33)		
	NCON	-		-		22.33 (0.15)		22.60 (0.13)		
DEC	UCL	23.01 (0.18)		23.45 (0.18)		22.86 (0.30)		23.34 (0.25)		
	Cup	22.97 (0.30)		22.88 (0.25)		22.66 (0.47)		22.21 (0.44)		
	League	22.74 (0.21)		22.77 (0.20)		22.46 (0.36)		22.31 (0.34)		
	NCON	-		-		22.66 (0.15)		22.90 (0.13)		
ACC_AVG_	UCL	0.73 (0.01)		0.74 (0.01)		0.75 (0.01)		0.76 (0.01)		c*
	Cup	0.74 (0.01)		0.76 (0.01)		0.77 (0.01)		0.80 (0.01)		c**, e*
	League	0.74 (0.01)		0.75 (0.01)		0.77 (0.01)		0.79 (0.01)		c***, e*
	NCON	-		-		0.75 (0.01)		0.77 (0.01)		f***
DEC_AVG_	UCL	− 0.00 (0.00)		− 0.02 (0.00)		− 0.02 (0.00)		− 0.06 (0.00)		c***, e***, f***
	Cup	− 0.00 (0.00)		− 0.02 (0.00)		− 0.02 (0.01)		− 0.03 (0.01)		
	League	− 0.00 (0.00)		− 0.02 (0.00)		− 0.03 (0.00)		− 0.05 (0.00)		c***, e***
	NCON	-		-		− 0.01 (0.00)		− 0.05 (0.00)		f***
Max. Speed	UCL	31.10 (0.16)		30.66 (0.16)		30.05 (0.23)	ø**	29.29 (0.20)		b***, c***, e***
	Cup	31.01 (0.23)		30.67 (0.20)		30.27 (0.34)		29.60 (0.32)		c**
	League	31.13 (0.18)		30.59 (0.17)		30.49 (0.27)		28.82 (0.26)		c***, e***, f***
	NCON	-		-		30.99 (0.15)	#**	29.42 (0.14)		f***
Nº Sprint	UCL	0.22 (0.01)		0.24 (0.01)		0.23 (0.01)		0.25 (0.01)		
	Cup	0.24 (0.01)		0.24 (0.01)		0.25 (0.01)		0.29 (0.01)		
	League	0.23 (0.01)		0.23 (0.01)		0.24 (0.02)		0.26 (0.01)		
	NCON	-		-		0.23 (0.01)		0.26 (0.01)		f***
HMLD	UCL	30.34 (0.59)		32.14 (0.58)		31.92 (0.78)		33.40 (0.69)	+**	a*, c***
	Cup	31.81 (0.77)		32.81 (0.70)		33.63 (1.09)		37.32 (1.05)	#**	c***, e**
	League	30.80 (0.63)		31.85 (0.61)		34.06 (0.89)		34.68 (0.85)		b*, c***, e*
	NCON	-		-		31.62 (0.55)		34.28 (0.52)		f***

Coeff = Coefficient, SE = Standard Error, Starters = players who played more than 150 min, Replaced = players who played between 95–149 min, Fringe = players who played between 60–94 min, Non-Starters = players who played between 0–59 min, ACC = number of total high accelerations per minute, DEC = number of total high decelerations per minute, ACC_AVG_ = average intensity of the accelerations performed in the match, measured in m/s^2^, DEC_AVG_ = average intensity of the decelerations performed in the match, measured in m/s^2^, Max. Speed = maximum speed achieved in the match, Nº Sprint = number of sprints performed per minute (> 24 km·h^− 1^), HMLD = distance covered with a power consumption above 25.5 W·kg-1 per minute, UCL = congestion caused by Champions League match, Cup = congestion caused by Cup match, League = congestion caused by League match, NCON = week in which only one match is played, non-congested week, # = significant differences respect to UCL; + = significant differences respect to Cup; µ = significant differences respect to League; ø = significant differences respect to NCON; a= significant differences between Starters and Replaced; b = significant differences between Starters and Fringe; c = significant differences between Starters and Non-Starters; d = significant differences between Replaced and Fringe; e = significant differences between Replaced and Non-Starters; f = significant differences between Fringe and Non-Starters; *p < .05; **p < .01; ***p < .001

Maximum running speed (Max. Speed): In NCON weeks, Fringe players reached significantly higher values compared to CON weeks by UCL (*p* < .01).

High Metabolic Load Distance per minute (HMLD): During CON weeks by Cup, Non-Starters players achieved significantly higher HMLD values than in UCL weeks.

No group of players experienced significant differences in ACC, DEC, DEC_AVG_ and Nº Sprint between competition types.

Between players comparison: In UCL congestion weeks, DEC_AVG_ was significantly higher for Starters players (*p* < .001). In Cup congestion weeks, HMLD was significantly higher in Non-Starters players than in Starters (*p* < .001) and Replaced players (*p* < .01). In League congestion weeks, ACC_AVG_, DEC_AVG_ and HMLD were significantly higher in Non-Starters players compared to Starters and Replaced players. Conversely, Max. Speed achieved was significantly lower in Non-Starters players compared to the rest (*p* < .001). Finally, during NCON weeks, Non-Starters players presented significant differences in ACC_AVG_, DEC_AVG_, Max. Speed, Nº Sprint and HMLD compared to Fringe players. Specifically, Non-Starters players achieved higher values in all these variables except in Max. Speed.

These results show that Non-Starters tend to have higher neuromuscular loads (ACC, DEC, HMLD) but lower peak speeds (Max. Speed) compared to Starters, Replaced, and Fringe players, particularly during weeks CON by Cup.

## Discussion

This study aimed to examine the influence of competition type (i.e., Cup, League and UCL) and match playing time on external load during CON weeks in top five Spanish professional soccer teams. This is the first study to examine differences in match running performance between weeks with one or two matches in professional soccer, considering the competition type and the match playing time. The main study results highlighted that: (a) Players in NCON weeks covered significantly less distance at VLSR and LSR compared to CON weeks; (b) Players in CON weeks by League covered significantly higher TD compared to UCL congested weeks and NCON weeks. HMLD was significantly higher in CON weeks by Cup compared to UCL, League and NCON weeks; (c) Non-Starters players covered significantly higher VHSR in CON weeks by Cup than UCL congestion and NCON weeks; (d) during NCON weeks, Non-Starters players achieved significantly higher values in ACC_AVG_, DEC_AVG_, Nº Sprint and HMLD compared to Fringe players.

### The Influence of Congested Schedule on External Load

During CON weeks, players covered significantly greater distances at VLSR and LSR compared to NCON weeks. This finding is consistent with previous reports showing higher low-intensity running volumes during CON fixtures [7]. This may reflect a pacing strategy whereby players increase their walking and jogging volume to preserve their ability to perform high-intensity efforts in response to the contextual demands of the match [21]. In contrast, Julian et al. [3] reported in their systematic review that CON weeks were generally associated with reduced distances at low and moderate intensities. Differences between studies may relate to methodological and contextual factors, such as the operational definitions of intensity thresholds, competition level, match context, and the specific congested patterns analysed. Notably, the absence of significant differences in TD in the present study does not preclude changes in the distribution of running across speed zones. Overall, these results support the need to carefully monitor external load and recovery management during periods of congestion to reduce the risk of injury in elite soccer [9].

### Differences on External Load According to Competition Type

Considering the competition type, the results showed lower values on TD, VLSR, LSR and MSR during CON weeks by UCL, compared to weeks congested by Cup, League or NCON weeks. This difference could be attributed to the higher demands posed by UCL opposition. In contrast, in the League and Cup, the lower level of opponents allows teams to use pacing strategies, which results in reduced fatigue. In terms of high intensity, during the CON weeks by UCL and League, HSR, VHSR and Sprint values were significantly lower than those of the CON weeks by Cup, where Spanish teams tend to implement more extensive squad rotation [22, 23]. Furthermore, inadequate hamstring recovery during congested fixtures, coupled with few lineup changes in order to win the match against extremely high-level teams, could further compromise performance in high-intensity actions [9]. In contrast, ACC and DEC showed significantly higher values during CON weeks by UCL, compared to weeks congested by League and NCON. This change could be due to different contextual or tactical variables (e.g., match dynamics, opponents’ approach, effective playing time, etc.). In turn, as mentioned above, this could be due to incomplete recovery (72 h) of the hamstring muscle structure, which makes it difficult for players to exert themselves at speeds above 24 km·h^− 1^ [24]. Finally, similarly to high sprint distances, during the CON weeks by Cup, HMLD values was significantly higher than those of the CON weeks by UCL, League and NCON weeks. Although these results differ from those observed in the ACC and DEC variables, could be due to the high sensitivity to change presented by the HMLD variable. In other words, HMLD records a range of accelerations of 2–4 m·s^− 2^ and speeds greater than 19.8 km·h^− 1^, so accelerations can be performed that are not counted in the ACC/DEC variables because they are less than 3 m·s^− 2^, but would be counted in the HMLD variable. For example, this can occur when players are running at high speeds and accelerate. It is difficult for them to reach > 3 m·s^− 2^, but they can reach > 2 m·s^− 2^, which is counted within HMLD.

### Differences on External Load according to Competition Type and Match Playing Time

The format of each competition can influence physical performance variables [25]. Cup and UCL are competitions that include knockout rounds, which may result in increased uncertainty and, consequently, the effort required to advance to the next round [26]. Additionally, the different structure of the UCL also affects external load variables such as TD, VHSR or Sprint [27]. Another fundamental aspect is the level of the opposition. Matches against stronger teams tend to elicit higher physical performance, particularly in the later stages of the Cup or UCL [28]. In some cases, the high workload performed by the player leads to greater fatigue accumulation, preventing them from having sufficient time for full recovery before the weekend league match [29]. Substitutions also play a role, as tactical decisions based on match outcome can modulate physical output [30]. Therefore, type of competition and match playing time are influential factors that could affect external load metrics and should be considered when interpreting physical performance in elite soccer.

The external load of Non-Starters players warrants particular attention, as it revealed significant differences compared to Replaced and Fringe players. Specifically, Non-Starters players covered greater distances at VHSR, demonstrated higher average accelerations and decelerations, accumulated greater HMLD, and a greater number of sprints during CON weeks by Cup matches compared to UCL and NCON matches. This could be because reduced participation in matches leads to players with fewer minutes of play exhibiting lower external loads [31]. Consequently, these players accumulate less fatigue and can perform at higher intensity during their minutes of participation in the weekend league [32]. Previous research has identified that players’ pacing behaviour can be influenced by their knowledge of exercise duration. For example, Pan et al. [13], demonstrated that in Chinese Super Soccer League substitute players covered significantly higher high-intensity running distance and sprint distance than replaced and entire match players. Therefore, the pacing strategies of substitute players may differ from those of starting players who complete the full match, as substitutes are fully aware of the time frame available for their performance [33].

Finally, the most relevant insights concerning competition type and player status indicate a decrease in high-intensity distance variables, such as VHSR during weeks CON by UCL compared to weeks CON by Cup matches, specifically among Starters, Replaced and Fringe when compared to Non-Starters players. One possible explanation is that the physical demands of the UCL may be greater than those of the Cup, leading players to accumulate higher levels of fatigue in weeks when UCL matches are played, which in turn could reduce their performance during the weekend league fixtures [34, 35]. Furthermore, player rotations implemented during Cup matches, as a consequence of the opponent’s strength, may also contribute to allowing several players to rest, enabling them to be in a better physical condition during league matches, as reflected by higher external load outputs [36].

### Limitations and Future Directions

This study has limitations that should be acknowledged. First, only Spanish teams were included in the sample, and therefore, the findings may not be generalisable to other professional leagues. Nonetheless, this study opens a new line of research by highlighting the relevance of player status in relation to competition type. A methodological consideration of this study concerns the operationalisation of match participation using categorical exposure profiles. Although this approach was based on previous literature and selected for its applied relevance, alternative analytical strategies could treat match playing time as a continuous variable to further explore dose–response relationships. Future studies may benefit from combining both approaches to refine the interpretation of external load responses during congested schedules. Another limitation lies in the exclusive focus on external load metrics. Relevant internal load indicators such as heart rate or perceived exertion along with tactical, technical, and psychological variables were not assessed, despite their potential influence on performance. Likewise, team planning and recovery strategies were not taken into account, which may vary each week depending on the type of competition played, the time available between matches, or their own interests. Additionally, GPS-based data do not capture contextual information such as opponent strength, environmental conditions, or team strategies, which may limit the interpretation of observed differences. Future studies may benefit from combining both approaches to refine the interpretation of external load responses during congested schedules. Future research should explore the role of injury incidence throughout the season, as fluctuations in match intensity may be related to overload-related injuries [37]. More comprehensive approaches should also consider match outcomes and the competitive ranking of opponents. Finally, comparative analyses of team performance across different competition phases (e.g., group stages vs. knockout rounds) could further enrich our understanding of external load dynamics.

## Conclusion

The present findings suggest that the type of competition included in CON weeks influences players’ physical performance, which varies according to individual playing time. Generally, players in NCON weeks covered significantly less distance only at low-intensity compared to CON weeks. In CON weeks by League matches, players ran greater total distances than in weeks congested with UCL or NCON matches. Specifically, Non-Starters players exhibited higher VHSR values in weeks when the Cup was played, compared to weeks congested by UCL or NCON weeks.

### Practical Applications

This study emphasises the need for close collaboration between coaches, sports scientists, and strength and conditioning professionals to optimise physical performance in top Spanish soccer teams. Additionally, the present research reinforces the importance of individualising training loads based on players’ exposure time to matches and the type of competition through top-up sessions. These results indicate that, for instance, players who accumulate more minutes show signs of fatigue when attempting to run at speeds of 18–21 km·h^− 1^. Therefore, it would be advisable to establish recovery strategies that help players prepare in the best possible condition for the next match. On the other hand, based on Soler et al. [38], preventive strategies focusing on calf muscles could be beneficial for Starters players after CON weeks by UCL, due to the increase of ACC and DEC efforts during these weeks. This approach could contribute to maintaining physical performance and preventing overtraining or fatigue.

## Supplementary Information

Supplementary Material 1.

## Data Availability

The data that support the findings of this study are available from https://www.laliga.es/ but restrictions apply to the availability of these data, which were used under license for the current study, and so are not publicly available. Data are however available from the authors upon reasonable request and with permission of LaLiga.

## Abbreviations

UCL: Champions League
Cup: National Cup
LaLiga: League
NCON: Non-congested
CON: Congested
TD: Total distance covered
VLSR: Very low-speed running
LSR: Low-speed running
MSR: Medium-speed running
HSR: High-speed running
VHSR: Very high-speed running
ACC: Number of high accelerations
DEC: Number of high decelerations
ACC_AVG_: Average intensity of the accelerations performed in the match
DEC_AVG_: Average intensity of the decelerations performed in the match
Max. Speed: Maximum speed achieved in the match
Nº Sprint: Number of sprints above > 24 km·h^− 1^
HMLD: Distance covered with a power consumption above 25.5 W·kg^− 1^
LMMs: Linear Mixed Models
Coeff: Coefficient
SE: Standard errors
