# Supplementary material for: Do the Competitions Played During Congested Weeks Influence the External Load of Spanish Soccer Teams? Analysis by Match Playing Time
Source: Sports Med Open. 2026 Mar 18;12:33. doi: 10.1186/s40798-026-01008-x (PMC13000035; doi:10.1186/s40798-026-01008-x)
Supplement: Supplementary file 1 — Supplementary Material 1. [file 40798_2026_1008_MOESM1_ESM.docx]

**Table 5**

Descriptive statistics of distance related variables in CON and NCON weeks (*Mean ± SD*).

|  | **TD**  **(m·min^-1^)** | **VLSR**  **(m·min^-1^)** | **LSR**  **(m·min^-1^)** | **MSR**  **(m·min^-1^)** | **HSR**  **(m·min^-1^)** | **VHSR**  **(m·min^-1^)** | **Sprint**  **(m·min^-1^)** | **High Sprint**  **(m·min^-1^)** |
| --- | --- | --- | --- | --- | --- | --- | --- | --- |
|  | Mean ± SD | Mean ± SD | Mean ± SD | Mean ± SD | Mean ± SD | Mean ± SD | Mean ± SD | Mean ± SD |
| **CON** | 120.97 ± 11.49 | 35.68 ± 6.22 | 40.29 ± 7.71 | 32.55 ± 8.25 | 8.01 ± 2.61 | 4.56 ± 1.79 | 2.72 ± 1.46 | 0.86 ± 0.89 |
| **NCON** | 120.36 ± 13.09 | 35.21 ± 4.15 | 39.77 ± 6.36 | 32.36 ± 7.99 | 7.98 ± 2.82 | 4.58 ± 2.04 | 2.81 ± 1.78 | 0.89 ± 0.96 |

*Note*. SD = Standard Deviation; CON = Congested week; NCON = Non-congested week; TD = Total distance; VLSR = distance covered between 0-6 km·h^-1^; LSR = distance covered between 6-12 km·h^-1^; MSR = distance covered between 12-18 km·h^-1^; HSR = distance covered between 18-21 km·h^-1^; VHSR = distance covered between 21-24 km·h^-1^; Sprint = distance covered between 24-28 km·h^-1^; High Sprint = distance covered above 28 km·h^-1^.

**Table 6**

Descriptive statistics of accelerations, decelerations and sprint variables in CON and NCON weeks (*Mean ± SD*).

|  | **ACC** | **DEC** | **ACC_AVG_** | **DEC_AVG_** | **Max. Speed** | **Nº Sprint** | **HMLD** |
| --- | --- | --- | --- | --- | --- | --- | --- |
|  | Mean ± SD | Mean ± SD | Mean ± SD | Mean ± SD | Mean ± SD | Mean ± SD | Mean ± SD |
| **CON** | 22.68 ± 3.18 | 23.01 ± 3.23 | 0.74 ± 0.09 | -0.73 ± 0.09 | 30.59 ± 2.17 | 0.23 ± 0.12 | 31.83 ± 7.10 |
| **NCON** | 22.62 ± 1.58 | 22.95 ± 1.63 | 0.73 ± 0.09 | -0.72 ± 0.09 | 30.60 ± 2.22 | 0.24 ± 0.13 | 31.76 ± 7.36 |

*Note*. SD = Standard Deviation; CON = Congested week; NCON = Non-congested week; ACC = number of total high accelerations per minute; DEC = number of total high decelerations per minute; ACC_AVG_ = average intensity of the accelerations performed in the match, measured in m·s^-2^; DEC_AVG_ = average intensity of the decelerations performed in the match, measured in m·s^-2^; Max. Speed = maximum speed achieved in the match; Nº Sprint = number of sprints performed per minute (> 24 km·h^-1^); HMLD = distance covered with a power consumption above 25.5 W·kg^-1^ per minute.

**Table 7**

Analysis of Intraclass Correlation Coefficient (ICC) for distance related variables.

|  | **TD** | **VLSR** | **LSR** | **MSR** | **HSR** | **VHSR** | **Sprint** | **High Sprint** |
| --- | --- | --- | --- | --- | --- | --- | --- | --- |
| **Fixed Intercept** | 121.49 | 35.16 | 40.28 | 32.94 | 8.14 | 4.71 | 2.86 | 0.88 |
| **Random Variance Player** | 85.88 | 8.94 | 19.37 | 39.37 | 3.68 | 2.04 | 1.36 | 0.33 |
| **Residual Variance** | 78.49 | 18.45 | 28.73 | 28.88 | 4.26 | 2.21 | 1.62 | 0.55 |
| **AIC** | 19,609.53 | 15,653.45 | 16,871.27 | 19,965.66 | 11,801.87 | 10,055.46 | 9,223.62 | 6,285.17 |
| **BIC** | 19,627.20 | 15,671.12 | 16,888.95 | 16,983.33 | 11,819.54 | 10,073.13 | 9,241.29 | 6,302.85 |
| **ICC** | 0.52 | 0.33 | 0.40 | 0.57 | 0.46 | 0.48 | 0.46 | 0.38 |

*Note.* AIC = Akaike Information Criterion; BIC = Bayesian Information Criterion; ICC = Intraclass Correlation Coefficient; TD = Total distance; VLSR = distance covered between 0-6 km·h^-1^; LSR = distance covered between 6-12 km·h^-1^; MSR = distance covered between 12-18 km·h^-1^; HSR = distance covered between 18-21 km·h^-1^; VHSR = distance covered between 21-24 km·h^-1^; Sprint = distance covered between 24-28 km·h^-1^; High Sprint = distance covered above 28 km·h^-1^.

**Table 8**

Analysis of Intraclass Correlation Coefficient (ICC) for accelerations, decelerations and sprint variables.

|  | **ACC** | **DEC** | **ACC_AVG_** | **DEC_AVG_** | **Max. Speed** | **Nº Sprint** | **HMLD** |
| --- | --- | --- | --- | --- | --- | --- | --- |
| **Fixed Intercept** | 22.58 | 23.08 | 0.75 | 0.73 | 30.40 | 0.24 | 32.41 |
| **Random Variance Player** | 1.52 | 1.92 | 0.00 | 0.01 | 1.81 | 0.01 | 30.34 |
| **Residual Variance** | 5.70 | 5.04 | 0.00 | 0.00 | 3.12 | 0.01 | 27.64 |
| **AIC** | 12,365.44 | 12,565.44 | -6,743.99 | -6,753.99 | 10,925.47 | -4.859.76 | 15,823.20 |
| **BIC** | 12,383.11 | 12,583.12 | -6,726.32 | -6.736.32 | 10,943.14 | -4.842.09 | 16,840.87 |
| **ICC** | 0.21 | 0.27 | 0.56 | 0.56 | 0.37 | 0.52 | 0.52 |

*Note.* AIC = Akaike Information Criterion; BIC = Bayesian Information Criterion; ICC = Intraclass Correlation Coefficient; ACC = number of total high accelerations per minute; DEC = number of total high decelerations per minute; ACC_AVG_ = average intensity of the accelerations performed in the match, measured in m·s^-2^; DEC_AVG_ = average intensity of the decelerations performed in the match, measured in m·s^-2^; Max. Speed = maximum speed achieved in the match; Nº Sprint = number of sprints performed per minute (> 24 km·h^-1^); HMLD = distance covered with a power consumption above 25.5 W·kg^-1^ per minute.
